# Navigating the Role of Radiotherapy for Extremity Soft Tissue Sarcomas

**DOI:** 10.3390/biomedicines14081744

**Published:** 2026-08-02

**Authors:** Emmy Nyqvist, Christina Linder-Stragliotto, Jenny Lofgren, Panagiotis Tsagkozis

**Affiliations:** 1 Department of Molecular Medicine and Surgery, Karolinska Institute, 17176 Stockholm, Sweden; christina.linder.stragliotto@ki.se (C.L.-S.); jenny.lofgren@ki.se (J.L.); panagiotis.tsagkozis@ki.se (P.T.); 2 Department of Reconstructive Orthopaedics, Karolinska University Hospital, 17176 Stockholm, Sweden; 3 Department of Breast, Endocrine and Sarcoma Oncology, Karolinska University Hospital, 17176 Stockholm, Sweden; 4 Department of Reconstructive Plastic Surgery, Karolinska University Hospital, 17176 Stockholm, Sweden

**Keywords:** soft tissue sarcoma, radiation, surgery

## Abstract

**Background/Objectives**: The basis for treatment of sarcoma is surgical resection. However, since the introduction of limb salvage surgery, guidelines recommend radiation as a complement to surgery for high-grade sarcomas. The aim of the present narrative review was to provide a comprehensive update about the role of surgery and radiotherapy (RT) for extremity soft tissue sarcoma (ESTS), to increase both surgeons’ and oncologists’ understanding of radiation as an adjuvant to sarcoma surgery and to illuminate areas of uncertainty that requires further research. **Methods**: A search on PubMed using relevant MeSH terms was performed, focusing on original research, meta-analyses and review articles. Related articles were identified through snowballing. **Results**: The support for the use of radiotherapy in conjunction with limb salvage is strong. Complication profiles differ between pre- and postoperative RT and attempts to further limit these are both technological and surgical. Preoperative radiotherapy provides the best results regarding long-term complications so far. Hypo-fractionation is showing promise, but awaiting long-term results for complications remains an area of research rather than clinical practice. Particle beam therapy and internal radiation show effects on control, but due to technical and availability issues, their role is mainly in previously irradiated or inoperable tumors. **Conclusions**: Upcoming research into adjusted deliveries, doses and planning is anticipated. To further personalize treatment, studies into sarcoma subtypes are indicated.

## 1. Introduction

Sarcomas are rare malignancies of supportive tissue with approximately 50 different subtypes of soft tissue sarcoma (STS) inhabiting somewhat different growth patterns. However, in research they are commonly grouped due to similar presentation and surgical treatment. Extremity soft tissue sarcoma (ESTS) makes up approximately 60%, with trunk and retroperitoneum being other common tumor sites. Grading of the tumor is done according to the FNCLCC system, but in most research simplified into low-grade and high-grade. In low-grade tumors wide resection alone provides good local control [1]. Surgical resection with wide margin is the mainstay of any curative attempt also for high-grade tumors with the benefit of additional radiotherapy, and sometimes chemotherapy, depending on multiple factors. Typical factors are tumor depth and size, where previous studies can show a benefit to local control with the addition of radiotherapy (RT) [2]. The role of RT in treatment for ESTS is vastly studied, but study designs and small populations limit the possibility to draw certain conclusions. Controversy still remains regarding timing of RT related to surgery, total dose and fractionation, as well as benefits of certain more recent technologies such as image guided and intensity modulated radiotherapy. The aim of the present review is to provide a comprehensive understanding of the mechanism of and role for radiotherapy and surgery in sarcoma of the extremities.

## 2. Search Strategy

A literature search was conducted to identify relevant publications for this review. Searches were performed on PubMed using MeSH terms [soft tissue sarcoma] AND [radiation OR radiotherapy] (Title) and [extremity] (all). The time period was set to 1982- September 2025, and only articles published in English were included. Inclusion criteria were original research and review articles, as well as international guidelines on the topic of extremity soft tissue sarcoma and radiotherapy. Articles presenting data on combined oncologic treatment, diagnostic methods or technical information without patient outcomes were excluded. A total of 260 titles were screened, and publications were excluded based on the use of other oncological treatments (*n* = 45) or the topic falling outside the scope of this review (*n* = 67). Letters to the editor or expert opinions were also excluded (*n* = 15), resulting in remaining 133 titles. From these articles, related research was identified through “snowballing” or “citation tracking”. The format of narrative review was chosen to allow for a broader summary of the research surrounding ESTS and RT to both provide a summary of current knowledge, and to identify areas for future research [3]. Where there are similar studies with similar results, only the most recent or the strongest evidence (randomized design or large population) is cited.

All registered studies on sarcoma and radiation were identified on clinicaltrials.gov. Ongoing and closed but not yet published studies are also presented. Various methods of radiotherapy (conventional external beam, intensity modulated and image guided, heavy ion particle, volumetric modulated arc therapy) are presented and their indications and results as compared to local control of the disease and patient survival are presented. Local complications and side effects are also discussed.

## 3. Results

### 3.1. Reasons for Radiotherapy in Limb Salvage Surgery

The primary treatment for all sarcomas is surgical resection with a margin of presumed healthy tissue. Historically, amputation was the main surgical option for local control in high-grade ESTS. In a ground-breaking study Rosenberg et al. [4] randomized 43 participants who received chemotherapy and either amputation or surgical resection with limb salvage, followed by adjuvant radiation. Similar results of local control were achieved for amputation versus limb salvage surgery (LSS) in combination with radiotherapy. LSS is now the gold standard for STS surgery.

Surgical principles for high-grade tumors are based on the Enneking system [5], in which the tumor’s relationship to fascial planes within a compartment determines the borders of resection. An intra-muscular tumor that is resected with intact muscle fascia as an outer border is considered to have wide margins. It has been consistently shown since the introduction of limb salvage surgery that tumor grade and surgical margins are the most important factor for local control [6,7,8,9].

If a resection is done with positive margins, re-resection should primarily be considered [10]. If this is not possible due to critical structures, radiation can be used as an adjuvant to reduce local recurrence (LR) [11]. Local control for this group will however not reach that of negative margins [12]. A Scandinavian registry-based study [13] showed that when surgical margins were positive (intralesional) or close but negative (marginal), the risk of LR was 39%. If postoperative radiotherapy was administered the risk was reduced to 24%. Furthermore, in patients with LR, the second local recurrence rate was 50% if the first was resected with marginal margins but reduced to 28% if re-resection with wide margin or adjuvant RT was added [13].

To increase local control, radiation can be used as an additive to surgery for STS also if margins are negative [9,14,15]. A meta-analysis based on 17 studies, one of them randomized, calculated risk for LR after surgery for STS with or without RT [6]. The risk of LR was reduced for patients receiving RT (OR 0.49, *p* = 0.001), but no effect on survival could be seen for ESTS. Another Scandinavian retrospective study comparing the treatment during 1986–1991 to that of 1998–2005 demonstrated an increased use of RT (28% vs. 53%) and a concurrent decrease in LR from 27% to 15% without any increase in wide margin resection [16]. Similar improvements in 10-year local control could be seen in a more recent study on control after intralesional surgery, followed by re-resection ± radiation. Patients without RT had 38% LR and those receiving 50 Gy postop radiation had 15% [17]. Support for the role of RT to increase local control thus remains strong [6,9].

#### 3.1.1. External Beam Radiation

In sarcoma, the reigning modality is that of ionizing radiation, delivered through external beams (EBRT) in either waves or particles.

Conventional radiation therapy (CRT), a type of external beam photon therapy, is the most investigated for sarcoma. The method delivers radiation through multiple beams with fixed positions, all with the same intensity, making it hard to spare surrounding tissue or structures. The dose to surrounding tissue is of the limiting factors for conventional radiation, causing side effects in the short- and long term as discussed below.

The other form of EBRT is particle beams where the energy released are actual carbon or proton particles moving into, but not through, the intended target, thus not delivering an exit dose to surrounding tissue. This feature is the reason for particle beams to be used for tumors in the spine or pelvis where sensitive neural structures need to be protected. Carbon ions have enhanced biological effectivity compared to protons and are therefore being investigated for less radiosensitive tumors. Particle beam RT is more expensive and less utilized, and in many places not readily available. Depending on tumor size and location, the surrounding tissue does not always receive a lower dose with particles than photon RT. There are also no studies comparing oncological outcome after photon vs. particle beam RT. Overall, photons are still the recommendation for ESTS.

**Figure 1:**
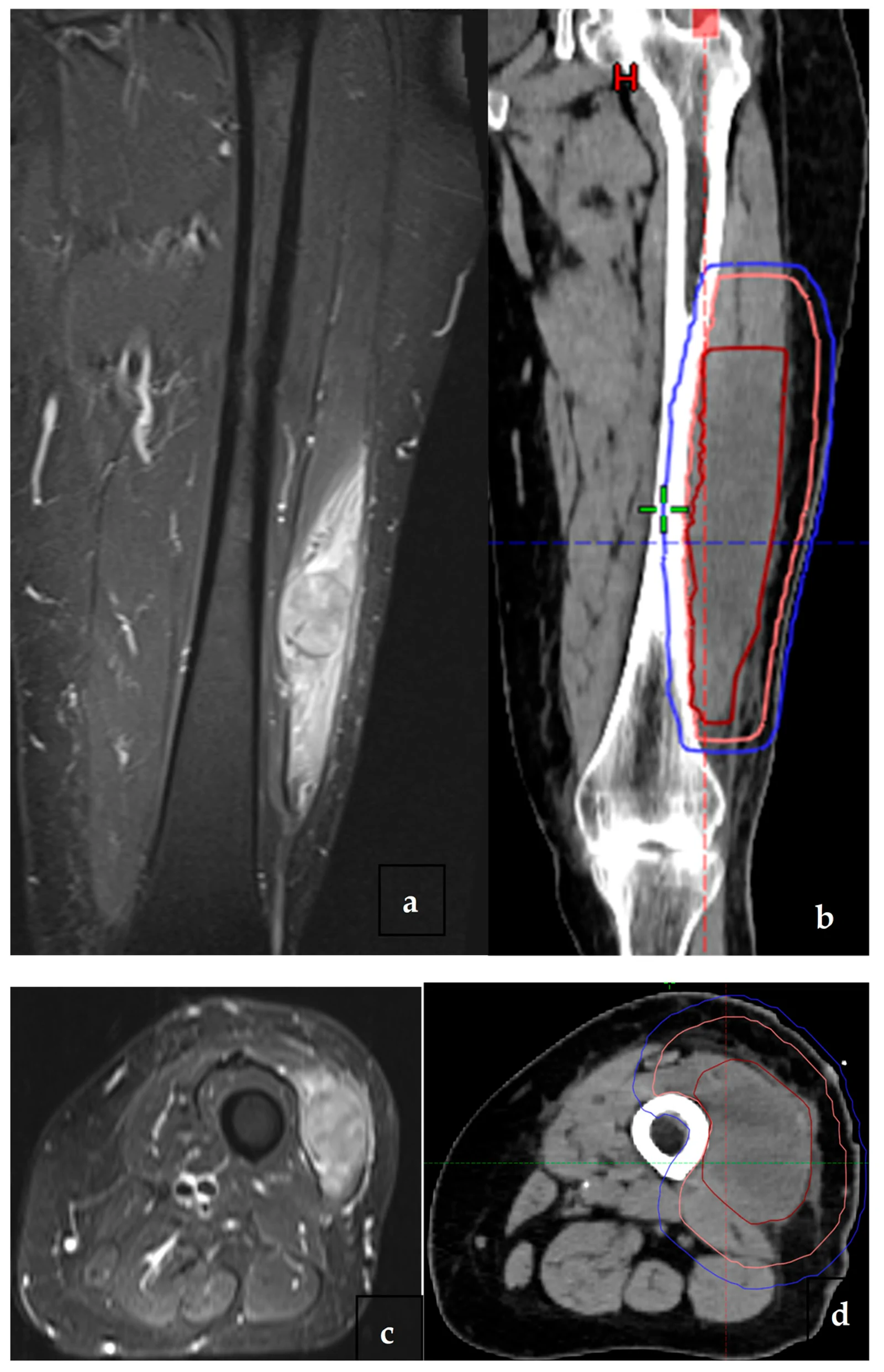
Imaging of a soft tissue sarcoma in the left thigh, illustrating preoperative tumor size and location as well as postoperative radiotherapy planning. (**a**,**c**) Preoperative MRI-imaging t1_tirm coronal and transverse of left thigh depicting a 26 × 30 mm STS in vastus lateralis. (**b**,**d**) Postoperative CT-imaging coronal and transverse with RT-fields drawn; GTV in red, CTV in pink and PTV in blue.

When planning for preoperative (neoadjuvant) RT in ESTS, the field is drawn onto imaging (usually CT and MRI). The gross tumor volume (GTV) is drawn from visible tumor. From this area, a safety margin is included to account for any tumor extension beyond the pseudo capsule. A small but significant study [18] indicating that sarcoma cells could be found up to 4 cm beyond the macroscopic tumor has influenced the size of this margin. A field of 0.5–1.5 cm radially and 3–4 cm longitudinally outside the GTV is commonly used, forming the clinical target volume (CTV). To adjust for movement or other inaccuracies, an additional 0.5–1 cm is added to all margins to form the planning target volume (PTV) [19,20] (Figure 1).

Even in postoperative RT the GTV is drawn from preoperative imaging. However, the CTV margins are expanded from a postoperative CT to include the whole surgical bed, the surgical scar and possible drain sites. These adjustments make the radiated field considerably larger compared to that of neoadjuvant RT. The total dose delivered to a deoxygenated and edematous postoperative tissue is also higher in postoperative RT.

Initially radiation was delivered postoperatively, but there are circumstances where preoperative radiation was thought to be beneficial, especially in cases where a possible size reduction could avert an amputation, or when wide resection is difficult due to proximity to large vessels or nerves. Even without a size reduction, if the tumor has been irradiated, a marginal resection reduces the risk of tumor contamination. On the other hand, if the tumor is resectable at diagnosis but any growth could risk resectability, immediate surgery with postoperative radiation is recommended. However, these clear-cut examples are often not common in ESTS. Most tumors could, technically, be irradiated either before or after surgery. Availability of either radiation or competent surgery varies throughout the world and will inevitably affect local tradition. One large, retrospective study on 1200 ESTS treated 1996–2010 showed better local control after preoperative as compared to postoperative RT [2]. In this study a majority of patients (66%) were treated postoperatively. Historic data like this reflect clinical practice before any randomized controlled trials (RCTs) were available. No differences in local control have been found based on timing in the major RCT on the subject since, but side effects differ [21].

Sometimes preoperative radiation is given with an added postoperative boost if margins are positive. However, in a retrospective study on 219 ESTS resected with positive margins after neoadjuvant RT 50 Gy, there was no proven benefit to adding such a postoperative boost (5-year local recurrence free survival (LRFS) 90% with vs. 74% without, *p* = 0.13) [22].

There is also the less common possibility to irradiate intraoperatively (IORT). This treatment sometimes replaces pre- or postoperative RT or can be used to boost their effect [23]. It can be delivered through brachytherapy or external beam therapy (explained below) and is more often used to spare organs at risk from radiation. However, in ESTS it is less common than, for example, in retroperitoneal sarcomas.

#### 3.1.2. Internal Radiation

Radiation can be delivered internally either into or surrounding the tumor or postoperative area. Brachytherapy (BRT) is the implantation of radioactive seeds into tissue, whereas trans-arterial radio-embolization is when radioactive beads are injected through a vessel feeding the tumor. These methods depend on the half-life of the implanted radioisotopes and the photon radiation of these releases. Neither technology is widely used in STS, and manual handling of the isotopes is labor intensive.

There have however been several publications on BRT showing an effect on local control [24,25]. Treatment can be before, during or after surgery, either as an inpatient or outpatient procedure. A benefit is that induction catheters can be placed at the time of surgical resection and treatment performed during the postoperative hospital stay, reducing the need for outpatient appointments for patients [26]. A retrospective study comparing 90 ESTS treated with either BRT, EBRT or a combination thereof indicated superior results for overall survival (OS) and LRFS for BRT [27]. However, the cohort consisted mostly of patients operated outside sarcoma centers (51%) and 14% already had local recurrence at the time of surgery. Other studies have shown BRT to have inferior local control (81% vs. 91% *p* = 0.04) compared to EBRT [28] in primary sarcoma. The American Brachytherapy Association’s recommendations suggest BRT primarily for recurrent STS or for lesions in previously irradiated areas [26].

### 3.2. Sarcoma Radiation Sensitivity

Soft tissue sarcoma is a heterogeneous collection of tumors. An early study on recurrence divided STS subtypes into spindle cell, pleomorphic, myxoid or round cell and showed very varied frequencies of LR (75% 33% 13% 0%), indicating the need for subtyping [29]. An experiment exposing 14 sarcoma cell lines in vitro to single RT doses 1–8 Gy calculated survival fractions [30]. Synovial sarcoma had the lowest survival fraction, indicating radio sensitivity, [30] but there was significant heterogeneity. One study retrospectively analyzed STS treated with neoadjuvant RT based on the four most common subtypes. In their data, no clear-cut differences in survival could be seen [31]. However, Myxofibrosarcomas (*n* = 19, 16% of population) tended to grow during treatment and RT did not affect recurrence rate or survival in this subgroup. Myxofibrosarcomas are infamously known to have infiltrative growth patterns, making resection with adequate margins difficult [32]. This is likely associated with the significant risk of recurrence of 20–60% and there is contention as to whether RT lowers this risk or not [7,33]. Myxoid liposarcoma is a subtype where we can see the clinical effect of RT on tumor size. Due to this clinical finding, the DOREMY [34] study investigated lowering the dose for preoperative RT in this sarcoma subtype and found no change in recurrence or survival. As a result, treatment can now be safely reduced. There are not many studies looking at sarcoma subtypes. Outside DOREMY, none are prospective and limited to the combination of radiotherapy and surgery, without other adjuvants.

It has been shown that oxygen is a modifier of radio sensitivity, and hypoxic tumor cells are less radio-responsive. After surgery the tissue in the wound area is also poorly oxygenated, thus requiring a higher dose of radiation.

#### Fractions and Dose

The total dose of radiation is delivered in fractions, the most common protocol for soft tissue sarcoma being fractions of 1.8–2 Gy to a total of 50 Gy. In postoperative RT a boost of 10–16 Gy is oftentimes added to the GTV, making the total dose 60–66 Gy. Hypo-fractionated RT (hypo-RT) is where each fraction is ≥2.2 Gy. With this method, the total dose can be delivered in fewer treatments which is convenient for the patient as well as the healthcare system.

Whether hypo-fractionation is beneficial to local control depends on the α/β-ratio of both the tumor and the organs at risk in the vicinity. The α/β-ratio is a measurement indicating the speed of cell proliferation and fraction sensitivity in a tissue. A low ratio indicates slower proliferation, less sensitivity to acute radiation toxicity and more late term radiation effect or toxicity. Tissues with a low ratio are more sensitive to hypo-fractionation, even when the total delivered dose is the same. The exact ratio for STS is not established but calculations are made from a presumed value of 3–5 Gy [35,36]. In a cell study the median α/β-ratio of 14 different sarcoma cell lines was 4.9 Gy, but 6 of the lines had a value below 4 Gy [30].

The biologically effective dose (BED) is calculated as a measure of true biological dose delivered by a particular combination of total dose and fraction size to a tissue with a specific α/β-ratio. Since almost all research into radiotherapy is based on 2 Gy fractions, other protocols can also be translated into equivalent dose in 2 Gy fractions (EQD2). Protocols with the same EQD2 can result in different BED depending on the tissue’s α/β-ratio (higher BED for a lower ratio and vice versa, see Table 1).

Lastly, if there is cell proliferation during treatment, the new cells receive only part of the total dose. This loss of effect can be beneficial to reduce toxicity of any organs at risk but can also limit the treatment effect if there is any pause throughout treatment [37]. If a protocol could be condensed the likelihood of disruption would be less, and this in our opinion provides another argument for hypo-RT.

### 3.3. Radiation-Induced Complications

When choosing whether to irradiate pre- or postoperatively, one needs to consider the complication profile of each. Grading of complication severity is done according to the European Organization for Research and Treatment of Cancer/Radiation Therapy Oncology Group criteria [38], a higher number indicating severity. The most common radiation-induced complication for ESTS is dermatitis. It is usually transient and can be treated locally, but if severe might hinder completion of the radiation protocol. In the case of preoperative RT, it could also delay the surgery. Most treatment protocols plan for surgery 4–8 weeks after preoperative RT to allow for delayed radiation effect to the tumor and reduced skin toxicity [39]. Similarly, a waiting period of 3–6 weeks is usually recommended after surgery to allow for wound healing before starting postoperative RT [40]. The risk of dermatitis is related to the administered dose of radiation, and since postoperative protocols oftentimes reach 60–66 Gy this side effect has been more common in adjuvant therapy. This is one of the suggested explanations as to why postoperative RT is less often completed, compared to preoperative (65% versus 77% *p* < 0.0001) [41].

In the short term, postoperative wound complications (WC) rather than radiation-induced dermatitis, have been identified as the most clinically relevant complication after limb salvage and RT. In the Canadian SR2 study, where 190 patients were randomized to either pre- or postoperative EBRT for ESTS, the risk of wound complication was greater (35% vs. 17% *p* = 0.01) for the preoperative group [21]. In a separate retrospective study from Canada, there was no difference in WC if surgery was performed anytime between 4 and 6 weeks after radiation commenced [42].

The association between WC and preoperative radiation is stronger in the lower limb [21]. These results have since been confirmed in retrospective studies [43,44] and the association is even stronger for the proximal limb [45]. Other known risk factors for surgical wound complications are tumor grade as well as size and depth (superficial tumors posing higher risk) [46,47]. Just as for other orthopaedic surgery, diabetes and smoking are also risk factors for postoperative wound issues [47,48]. There has been no prospective research into the relationship between radiation dose and surgical wound complications. Clinically, patients undergoing postoperative RT more often receive a higher total dose compared to preoperative RT, and still on average have less WC. In a small study on ESTS treated with neoadjuvant pencil beam proton radiation, 39% developed wound complications [49], which is in line with historic photon data.

The consequence of a WC can be repeated surgery, prolonged antibiotic treatment and multiple hospital visits for dressing changes. However strenuous for the patient and the healthcare provider, it is usually transient and will result in a healed wound and retained limb. However, there have been some studies indicating that the event of postoperative wound complication could influence oncological outcome. In one study the 5-year LRFS in patients that had a postoperative complication was 49% vs. 78% in the group without a complication [50]. Another study [51] showed a 5-year disease-specific survival (DSS) of 68% in patients with a postop complication vs. 81% in those without (*p* = 0.001). Both studies are retrospective, which introduces confounding, and further research into a possible link between WC and recurrent disease is warranted. Local factors such as immune modulation, inflammatory response as well as pure selection bias could possibly be involved.

Lymphedema following RT can be both acute and chronic and is graded 0–3 with increasing severity, according to ISL (international society of lymphology) [52]. To reduce the risk of lymphedema it is recommended to avoid circumferential radiation by sparing a soft tissue corridor [53]. Sparing a larger percentage of the extremity volume can result in less severe and less development of long-term oedema [54].

The most prevalent long-term complications after RT for STS are lymphedema, tissue fibrosis, joint stiffness or bone fracture. These symptoms can be progressive up until about 2 years post treatment, and in the case of fracture even later. The risk of fracture after BRT is reportedly 3–4% [24,55] and 2–4.5% after external beam radiation [56,57]. Fracture after EBRT relates to doses of radiation delivered to a long-bone circumference, and to whether the periosteum is stripped during surgery [57].

Soft-tissue fibrosis is much more common than fracture. The cohort from the prospective Canadian SR2 study was analyzed for radiation-related complications at 2 years and there was a non-significant difference in grade ≥2 fibrosis between postoperative compared to preoperative RT (48% vs. 32% *p* = 0.07). Field size was predictive of the development of fibrosis (*p* = 0.002) and stiffness (*p* = 0.006) [58]. If a tumor is large or close to sensitive structures, preoperative radiation should be considered to allow for a smaller radiation field and less fibrosis in adjacent tissue.

The IMRiS phase II trial on intensity modulated radiotherapy (IMRT) sought out to investigate fibrosis after RT and limb salvage [59]. In total, 159 patients were prospectively included and received either preoperative (67%) 50 Gy or postoperative (33%) 60–66 Gy in 2 Gy fractions. Out of 111 patients evaluated at 24 months, only 11% had grade ≥2 fibrosis which is considerably less than older technologies.

A feared long-term complication after radiation is that of secondary, radiation-induced malignancy. Sarcoma is one type of radiation-induced tumor, the presentation is usually many years after treatment and the prognosis is poor [60,61]. Radiation for other, more common malignancies such as breast- or prostate cancer and lymphoma is often the cause. No studies on risk for secondary malignancy after RT for sarcoma could be identified, likely due to the inherent rarity of the disease. Research into long-term effects of radiation for other malignancies can however be utilized to make assumptions on risk after RT for ESTS.

For example Brenner et al. [62] investigated second malignancies in patients treated for prostate cancer and found a significand risk increase of 15% after 5 years if treated with radiation compared to surgery. The risk difference was greater at 10 years reaching 34%. Presumably, the increased long-term survival after many cancers will give a rise in secondary, treatment-related malignancies. Reduction in radiation to healthy tissue must be a priority.

### 3.4. Adaptations to Reduce Complications

Novel RT methods have the potential to reduce complications. Volumetric modulated arc therapy (VMAT) is a type of EBRT where the radiation is delivered by a spinning source dispersing the rays through the surrounding tissue. VMAT technology can be used to deliver stereotactic body radiation therapy (SBRT) which is a form of treatment protocol utilizing high-dose fractions (hypo-RT) to treat small, well-circumscribed tumors (e.g., lung or spine) where precision is of utmost importance. A retrospective comparison of adjuvant CRT and VMAT in 109 patients with ESTS could show better bone-sparing using VMAT without compromising local control [63].

Intensity modulated radiotherapy (IMRT) is another type of EBRT utilizing fixed beams with different intensity, relying on complex computer calculations to adjust intensity. This allows treatment of more irregular tumors and better spares sensitive structures. The major argument for IMRT is the presumed reduced toxicity to healthy tissue [59]. A retrospective study on ESTS treated with IMRT or stereotactic body radiation therapy (SBRT), either pre- or postop LSS, could also show less LR (8% vs. 15% *p* = 0.05) for IMRT [64]. Despite patients having more close margin resections, IMRT remained a significant factor for reduced LR (HR 0.46 *p* = 0.02) in multivariate analysis, adjusting for tumor size [64]. Hence, there might be more benefits to IMRT than those of less local toxicity.

Image guided radiotherapy (IG-RT) refers to a more detailed imaging scheme and can be used in conjunction with other technologies. Planning is the same, but at the start of every treatment the extremity is imaged (oftentimes CT) and compared to the plan. If positioning or size has changed, adjustments are made. This method increases accuracy and reduces the size requirements of the PTV, hence reducing the irradiated area. In one study into neoadjuvant IG-IMRT the radiation field was drawn with consideration to future skin incisions, but the rate of WC was still comparable to traditional RT at 31% [65]. For STS, two randomized controlled studies on preoperative IG-RT in combination with either CRT or IMRT, each presenting more than 3 years follow-up data, showed reduced late toxicity to approximately 10% [65,66]. This contrasts with the 32% late toxicity in an earlier study on CRT alone [58]. The results for small, clearly defined tumors have not been superior to CRT.

There are some recent prospective studies into different fractionation protocols (Table 2), most presenting wound complication in 25–33% of cases [67,68,69,70,71,72], with one outlier having 41% [73]. In that study [73] patients underwent surgery within 7 days of finishing RT, whereas most other protocols suggest a surgical window 4–6 weeks after RT.

Two studies with the same hypo-RT protocol of 5 × 7 Gy [67,74] presented very different late toxicity, likely due to the short follow-up in one [74], resulting in an underestimation of true long-term complications.

Most hypo-RT series in STS still have short follow-up, but surprisingly the rate of fracture seems to be slightly higher than anticipated [69,76] within this time frame. Also worth noting is the abnormally high rate of amputations due to complications presented in the 8 Gy fraction series [71]. This suggests a limit to the hypo-RT tolerance that needs further investigation. Remembering that healthy tissue with a α/β-ratio of 2 will be more sensitive to the long-term effects of hypo-fractionation, reports on long-term follow-up are important. The total RT dose is also a risk factor for future radiation-induced malignancy, and such events occur in the very late setting. A recent consensus report recommended abstaining from hypo-RT in STS outside clinical trials until more data is available [77].

Multiple studies have investigated the effect of flap closure in ESTS to lessen WC, but results show no difference. In one series of 46 patients operated with LSS and flap reconstruction, radiation was still a risk factor for WC. In total, 14% of patients without and 50% of patients treated with RT experienced WC (*p* = 0.03) [78]. Comparison is difficult in retrospective material, and since most centers use flaps only when direct closure is not possible, bias is almost inevitable [79]. If the treatment area is close to skin, the wound can be temporarily closed during treatment and secondarily covered with a flap [28,80]. A team from Melbourne has increased the use of flaps beyond the need for coverage with the aim to achieve wound healing without complication. In their retrospective series of 122 cases of ESTS undergoing neoadjuvant radiation with 50.4 Gy, resection and flap reconstruction was evaluated [81]. A total of 25% of patients experienced major wound complication and 17% of patients needed additional surgery due to WC. Some of these complications were to donor sites where surgeries were simple, and when excluded there was only a 20% WC rate. This might be a good trade-off between reduced WC to resection site and some less complex WC at donor site. A study into donor site morbidity after harvesting fascia lata flap report acceptable complications such as soft tissue bulging and mild pain [82].

### 3.5. Survival

Despite radiation evidently providing good local control, there has been controversy whether this translates into improved survival. Historically no such association could be established, but two studies have been able to link RT to survival benefits. One large study on registry data analyzed 6960 patients with ESTS undergoing limb salvage and radiation between 1988 and 2005 [1]. In this material OS for high-grade tumors at 3 years was 73% in irradiated patients and 63% for the non-irradiated (*p* < 0.001). In a meta-analysis [43], data from six studies totaling 4192 participants were pooled and authors could show that postoperative radiation was associated with improved survival (OR 1.19 *p* = 0.01) and fewer wound complications. In a separate meta-analysis [83], 2 RCTs and 28 cohort studies were included, totaling 3027 patients. Adjusted HR for mortality was 0.65 [95% CI: 0.52–0.82] for STS treated with surgery and RT compared to surgery alone. Both meta-analyses were mainly based on retrospective studies, which can bring bias or confounders into play. However, such large populations are difficult to assemble otherwise. The addition of chemotherapy regiments in high-risk tumors has shown benefits for both recurrence and survival [84,85] but true randomization for the combination of radiation and chemotherapy is rare and falls outside the scope of this review.

### 3.6. Ongoing and Upcoming Studies

One upcoming study comparing different types of neoadjuvant particle radiation is underway [86]. The protocol will randomize ESTS between proton- and carbon RT, given as 39 Gy in 13 fractions with wound complications as the primary outcome, the hypothesis being that dose-sparing of skin and adjacent tissue would reduce WC while keeping local control. There are multiple similar studies into hypo-RT for photons that are ongoing, most of them single arm phase II (Table 3).

They investigate different hypo-RT protocols for preoperative EBRT with regard to wound complications, local control and late toxicity. There are a few exceptions to this design, among them the SCOPE trial from The Netherlands (NCT04425967), planning to compare preoperative EBRT in different fractionations in 168 patients. Initially 14 fractions of 3 Gy compared to the traditional 25 fractions of 2 Gy, and for SCOPESII (NCT07071727) the moderately hypo-RT protocol will in turn be compared to 5 fractions of 6 Gy.

One group from Wisconsin plan to compare preoperative IMRT for the traditional 25 fractions of 2 Gy to a short course of 5 fractions of 5.5 Gy (NCT05109494). Their protocol stands out due to the relatively small dose given in the short course; 27.5 Gy in 5 fractions equivalent to EQD2 of 43.5 Gy_4_. The primary outcome from this study is to be histological response to radiation, why surgery is scheduled already 1–2 weeks after RT is finalized.

One of the very few upcoming studies also investigating postoperative radiation has finalized inclusion. This is a multi-center phase III RCT run from Toronto, planned to include 210 patients, comparing preoperative and postoperative IG-IMRT for STS of extremity or trunk. Both arms deliver 50 Gy in 25 fractions, with the possibility to give postoperative boosts of 16 Gy for intralesional resections (NCT02565498).

## 4. Conclusions

Within the field of sarcoma in general there are few randomized controlled trials. The lack of high-level studies poses a problem to the development of clinical guidelines. The many single- or multi-center, retrospective series available tend to rehash what the limited prospective and even fewer randomized controlled studies have suggested we do. Some studies try to overcome some limitations of the retrospective study design by inverse probability matching or weighting. However, the multifaceted complexity of presentation; histological subtype, size, grade and anatomy is difficult to balance, especially in limited case series. In addition, some areas of research presented in this article were identified through citation tracking, and there is a risk that the representation of research is not complete. Also, not all articles identified are referenced; this is due to many publications presenting only small case series with limited generalizability.

Some areas have been repeatedly assessed, and evidence for the following is moderate. The risk of local recurrence in ESTS is decreased with all types or radiation, but more so with external beam therapy [28]. Depending on size, shape and anatomical location different modalities (particle beam, IMRT, VMAT) can be considered [49,63,64]. The rate of surgical wound complications differs between pre- and postoperative treatment [21], and can possibly be reduced further with the use of surgical flaps [81].

**Figure 2:**
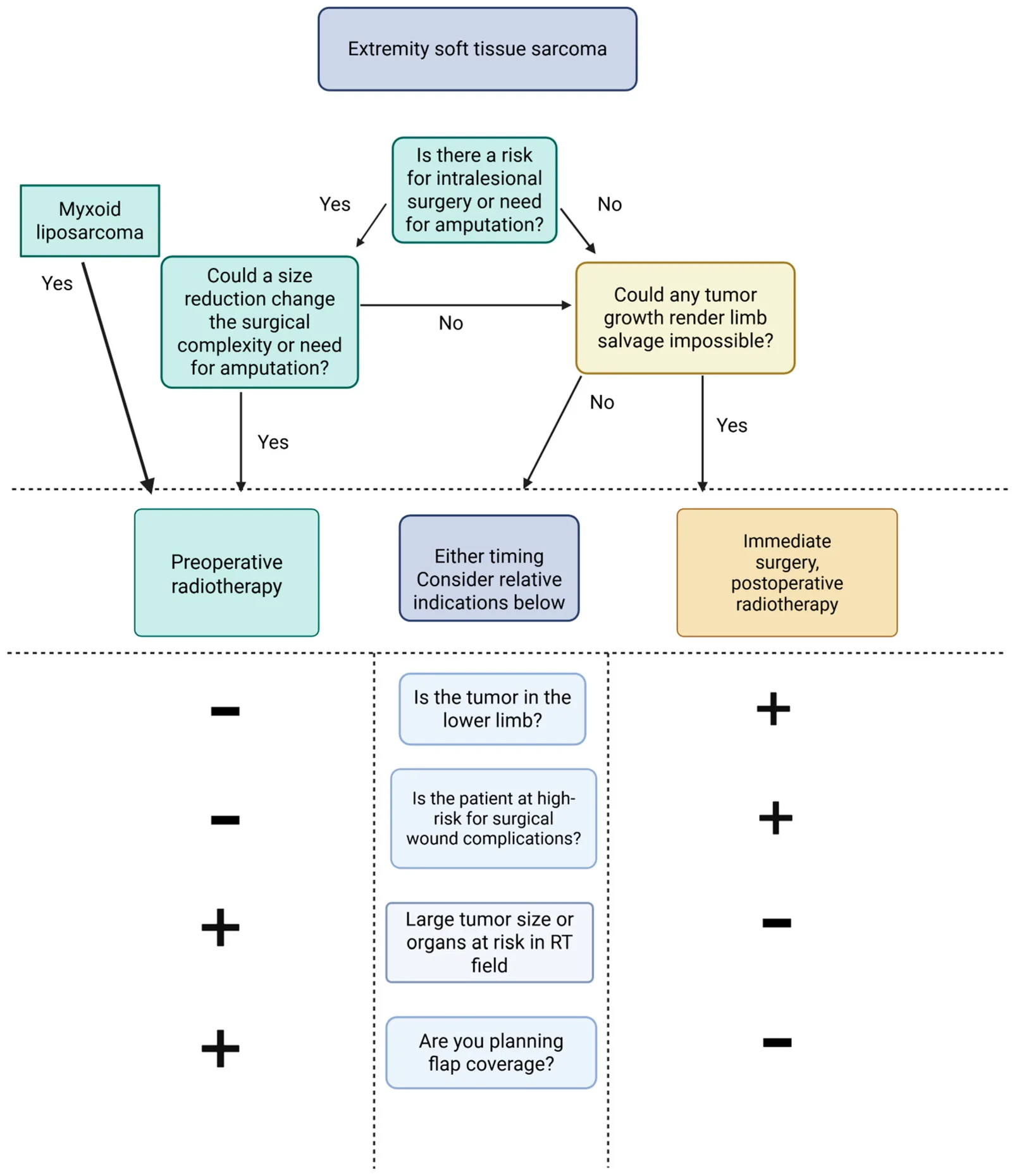
A flow chart for when to consider preoperative radiation or immediate surgery for soft tissue sarcoma. Created in BioRender. Tsagkozis, P. (2026) https://BioRender.com/d36on48 (accessed on 12 July 2026).

The field and dose of radiation have historically been larger when delivered postoperatively, something that is in turn linked to complications such as fibrosis and stiffness [58]. Current knowledge indicates that preoperative radiotherapy offers fewer long-term complications than postoperative RT and therefore should be the primary choice for most patients, the patient’s risk factors for surgical wound complications and the consequence of such a complication being factors favoring postoperative RT (Figure 2).

Modern techniques such as image guidance and intensity modulated radiotherapy make it possible to increase accuracy and reduce field size, allowing for better local control and less tissue toxicity [59,65,66].

The main indication for IG-RT or IMRT persists in areas with sensitive organs at risk close to the radiation field.

The total dose of radiation can be delivered in a shorter time frame through hypo-fractionation (Table 2), with promising results for local control (LC) and overall survival (OS). Awaiting reports on long-time follow-up regarding side effects, hypo-RT remains an area of research, and not yet a clinical recommendation.

There are a lot of questions in sarcoma research that are yet to be answered.

In large, pooled data-sets RT was linked to better survival, the ultimate goal of any oncological treatment. There is a remaining risk of confounding since most studies in this meta-analysis [43] were retrospective, and large prospective studies into this area are needed to reproduce this suggested link to survival.

## 5. Future Direction

Reductions in field and dose are necessary to overcome late toxicity. Interestingly, the way a field is drawn for EBRT does not take the Enneking principles into consideration. The CTV is drawn from calculations of distance to the tumor and will expand to cover scars and drains. Sometimes even if surgery was performed with negative margins and contamination throughout the scar should be exceedingly unusual. This is an area where surgical and oncological principles do not overlap. The postoperative anatomy can also be changed considerably, moving musculature from other compartments into the radiation field (Figure 1). Collaborations between surgeons and radiation oncologists could help identifying areas of future research, such as planning radiation fields.

The role of particle beam radiation in sarcoma needs to be further investigated, both in cases of inoperable tumors and as an adjuvant to surgery, the limiting factor being availability. Two upcoming registered studies will investigate definitive radiation with photons in hypo-RT protocols (NCT03972930, NCT06980259). This will only help a small portion of patients with access to particle radiation but could be of importance in cases of sensitive organs at risk in the field.

Spatially fractionated radiotherapy (SFRT) is an old idea made possible through new technology. The principle is that of delivering a high dose of radiation to only part of a bulky tumor such as sarcoma and sparing surrounding tissue. 3D technology (called lattice radiotherapy or LRT) and image guidance, as well as high-energy linear accelerators, has brought back the idea [87]. For sarcoma it is briefly investigated in a palliative setting [88] or for inoperable tumors [89], providing stable or reduced tumor size and subjective symptom relief. Protocols and indications need to be structurally investigated through future research. One study in China (NCT06980259) investigating definitive RT, including SFRT, is currently recruiting (Table 3).

Lastly, since we know that Myxoid Liposarcoma has a different response to RT [34], it is safe to assume this subtype is not the only outlier. The next step should be larger investigations on the major subtypes of STS. The most efficient treatment for each type is likely to differ due to different α/β-ratio, survival fractions, size, chemotherapy response and other factors yet unknown. An observational study out of France that has just completed inclusion of 300 patients (RADIOSARC, NCT05739084) was trying to identify genetic signatures of radio sensitivity among different types of STS. Larger studies, both clinical and molecular, focusing on each of the more common subtypes are necessary, striving toward a more personalized treatment protocol for extremity soft tissue sarcoma. Building a system where tumor- and patient-related risk factors for complications and predicted additive effect of radiation can be combined requires high-level evidence. Multi-center or even multi-national prospective studies would be required for large enough populations, but the possible gain cannot be exaggerated.

## Figures and Tables

**Table 1:** An overview of different radiation protocols studied for sarcoma, doses and fractions and their translation into biologically effective dose or equivalent dose in 2 Gy fractions.

Mode of Radiation	Total Dose (Gy)	Dose per Fraction (Gy)	BED_3_ (Gy)	BED_10_ (Gy)	EQD2_3_ (Gy)
CRT	50	2	83	60	50
IMRT	25	5	67	37.5	40
CRT	42	3	84	54.6	50.4
SBRT	35	7	117	59.5	70
IG-IMRT	40	8	147	72	88

CRT = conventional radiotherapy, IMRT = intensity modulated radiation therapy, SBRT = stereotactic body radiation therapy, IG-IMRT = image guided intensity modulated radiation therapy, BED_3_ = biologically effective dose in a tissue with α/β-ratio 3, BED_10_ = biologically effective dose in a tissue with α/β-ratio 10, $BED=D[1+\frac{d}{\alpha /\beta }]$ where D is total dose (Gy) and d is dose (Gy) per fraction, α/β for sarcoma commonly set to 3, EQD2_3_ = equivalent dose in 2 Gy fractions with α/β-ratio 3.

**Table 2:** Published hypo-fractionated radiation protocols for sarcoma and their reported outcome.

Study	Subjects	RT Modality	Field	Dose	Fractions	BED	EQD2	Time to Surgery	WC	LR	Late Complication	Follow Months	OS
IRB1302, Kubicek [74], 2018	14	SBRT	reference *	35–40 Gy	5 × 7 Gy	96 Gy_4_	64 Gy_4_	4–8 w	28%	7%	0	9	N/A
Bedi [67], 2022	32	IG-3dCRT	reference *	35 Gy	5 × 7 Gy	84 Gy_5_	60 Gy_5_	4–6 w	25%	0%	35% fibrosis	36	82% 3 year
HYPORT-STS Guadagnolo [69], 2022	120	3dCRT, IMRT, proton	Larger	42.75 Gy	15 × 2.85 Gy	73 Gy_4_	49 Gy_4_	4–6 w	31%	5%	2 fractures	24	N/A
Mayo [73], 2023	22	IMRT, VMAT	reference *	30 Gy	5 × 6 Gy	75 Gy_4_	50 Gy_4_	1–7 d	41%	0%	18%	25	91% 1 year 76% 2 year
Kalbasi [70], 2020	52	3dCRT, IMRT, electron beam	reference *	30 Gy	5 × 6 Gy	75 Gy_4_	50 Gy_4_	2–6 w	32%	6%	16%	29	84%
Gobo-Silva [68], 2021	10	3dCRT, IMRT	Larger	25 Gy	5 × 5 Gy	56 Gy_4_	35 Gy_4_	4–6 w	33%	5%	1 fracture, 1 amputation	29	DFS 72%
Kosela-Paterczyk [75], 2021	311	3dCRF, IMRT, VMAT	Larger	25 Gy	5 × 5 Gy	56 Gy_4_	35 Gy_4_	2–4 d	24%	14%	1 fibrosis, 3 fractures	N/A	63% 5 year
Leite [71], SABR, 2021	25	SABR by IG-IMRT	Smaller	40 Gy	5 × 8 Gy	146 Gy_3_	88 Gy_3_	med 60 d	28%	0%	4 amputations 1 fracture	21	N/A
Nikitas [72], 2025	110	IMRT, 3d-CRT	reference *	30 Gy	5 × 6 Gy	75 Gy_4_	50 Gy_4_	3–5 w	30%	8%	19% 5 amputations 3 fractures	37	85% 2 year

Late complication ≥ grade 2 of any kind. Where severe complications such as fracture or amputation were reported, these are presented as total number of cases; Field * reference is the one described in RTOG [75] of CTV = GTV + 1 cm radial and 2 cm longitudinal for low-grade tumors, and CTV = GTV + 1.5 cm radial and 3 cm longitudinal for high-grade tumors. PTV for all = CVT + 5 mm circumferential. Larger or smaller (than ref) = fields of different sizes than reference (as explained above); EQD2 = equivalent dose in 2 Gy fractions, IG-3dCRT = image guided 3D conventional radiation therapy, N/A = data not available/reported, DFS = disease free survival, SABR = stereotactic ablative radio therapy.

**Table 3:** A presentation of registered studies on clinicaltrials.gov on the subject of soft tissue sarcoma and radiation.

Clinical Trials Number	Subjects	Location	Status	RT Timing	RT	Arms	Dose	Fractions
NCT03819985	122	USA	active, not recruiting	Preoperative	EBRT	1	42.75 Gy	15 × 2.85 Gy
NCT04562480	120	USA	active, not recruiting	Preoperative	EBRT	1	42.75 Gy	15 × 2.85 Gy
NCT04425967	168	The Netherlands	active, not recruiting	Preoperative	EBRT	2	42 Gy vs. 50 Gy	14 × 3 Gy vs. 25 × 2 Gy
NCT07071727	150	The Netherlands	Recruiting	Preoperative	EBRT	2	42 Gy vs. 30 Gy	14 × 3 Gy vs. 5 × 6 Gy
NCT04506008	40	USA	Recruiting	Preoperative	EBRT	2	N/A	5 × N/A vs. 15 × N/A
NCT05938374	52	China	Closed	Preoperative	IMRT	1	43.5 Gy	15 × 2.9 Gy
NCT06087861	75	USA	Recruiting	Preoperative	EBRT	1	30 Gy	5 × 6 Gy
NCT06905132	40	USA	Recruiting	Preoperative	EBRT	1	30 Gy	5 × 6 Gy
NCT06022159	48	France	Recruiting	Preoperative	IMRT	1	30 Gy	5 × 6 Gy
NCT04330456	30	Russia	Recruiting	Pre- and post	SBRT CFT	2	25 Gy SBRT 50 Gy CFT	5 × 5 Gy vs. 25 × 2 Gy
NCT05109494	30	USA	Recruiting	Preoperative	IMRT	2	50 Gy vs. 27.5 Gy	25 × 2 Gy vs. 5 × 5.5 Gy
NCT02565498	210	Canada, Belgium, USA	active, not recruiting	Pre- and post	IG-IMRT	2	50 Gy	25 × 2 Gy
NCT03972930	48	USA	Inclusion complete	Definitive RT		1	≤60 Gy	3–8 × N/A
NCT06980259	106	China	Recruiting	Definitive RT	CRT SFRT	2	≤60 Gy	15–20 × 3 Gy ± 3–4 × 8–15 Gy

Status = study status (active, recruiting, not recruiting, closed), RT timing = radiotherapy pre- or postoperative, RT = type of radio therapy, Arms = number of treatment arms in study, N/A = no available information, CFT = conventionally fractionated therapy, SFRT = spatially fractionated radiation therapy.

## Data Availability

No new data were created or analyzed in this study. Data sharing is not applicable.

## Abbreviations

The following abbreviations are used in this manuscript:

RT	Radiation therapy
ESTS	extremity soft tissue sarcoma
STS	soft tissue sarcoma
LSS	limb salvage surgery
LR	local recurrence
EBRT	external beam radio therapy
CRT	conventional radiation therapy
CT	computer tomography
MRI	magnetic resonance imaging
GTV	gross tumor volume
CTV	clinical target volume
PTV	planning target volume
RCT	Randomized controlled trial
LRFS	Local recurrence free survival
IORT	Intra operative radiation therapy
BRT	brachytherapy
OS	overall survival
Hypo-RT	hypo fractionated radio therapy
BED	biologically effective dose
EQD2	equivalent dose in 2 Gy fractions
SBRT	stereotactic body radiation therapy
IMRT	intensity modulated radio therapy
WC	Wound complication
DSS	disease specific survival
VMAT	volumetric modulated arc therapy
IG-RT	image guided radiation therapy
LC	local control
CFT	Conventionally fractionated therapy
SFRT	Spatially fractionated radiation therapy
